# Supplementary material for: Impact of Infection with Flavobacterium psychrophilum and Antimicrobial Treatment on the Intestinal Microbiota of Rainbow Trout
Source: Pathogens. 2023 Mar 14;12(3):454. doi: 10.3390/pathogens12030454 (PMC10055933; doi:10.3390/pathogens12030454)
Supplement: Supplementary file 1 [file pathogens-12-00454-s001.zip › pathogens-1998414-supplementary.pdf]

Supplemental Material

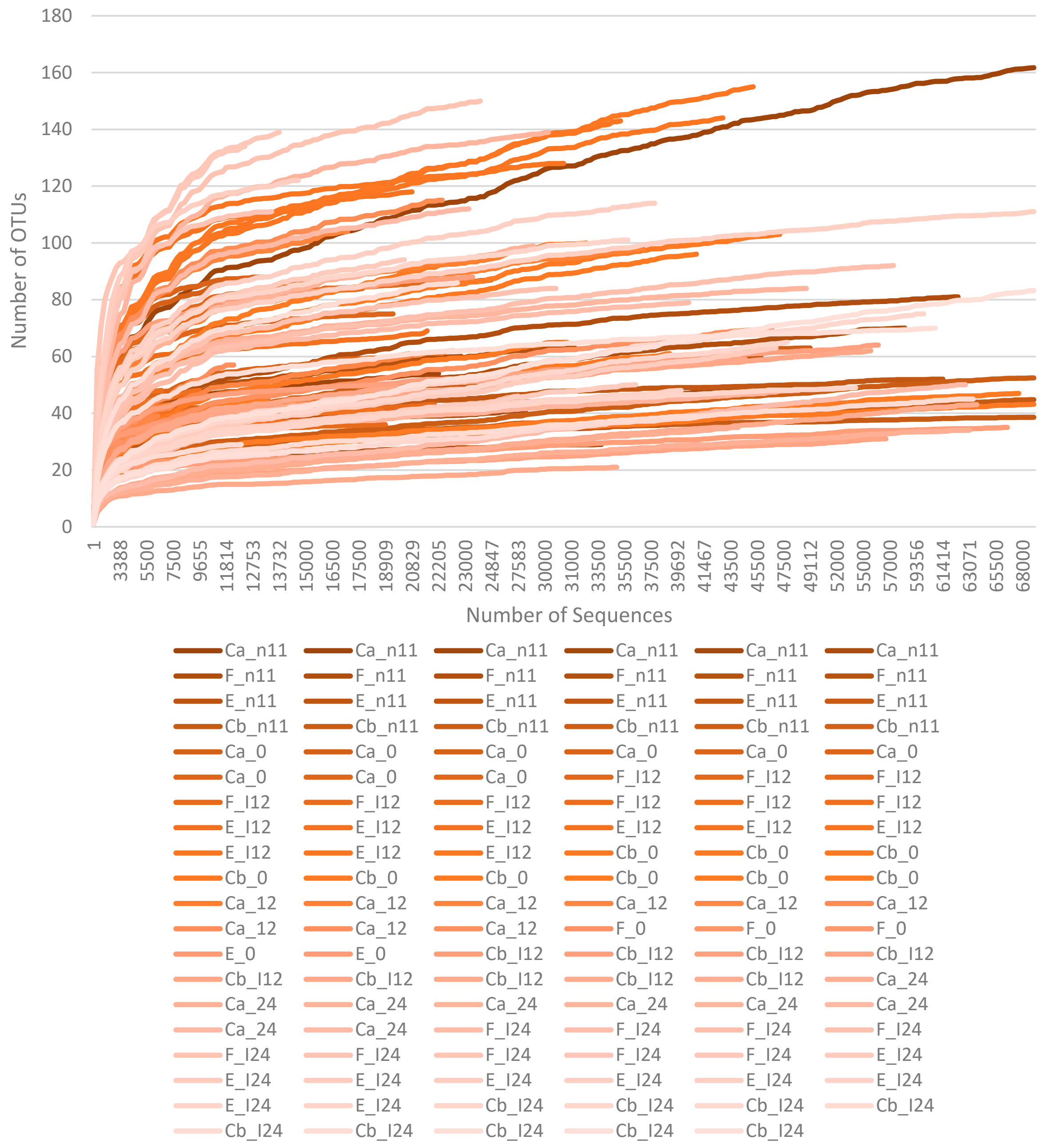

Figure S1. Rarefaction curve of each 16S sample from the intestinal content of rainbow trout.

(**a**)

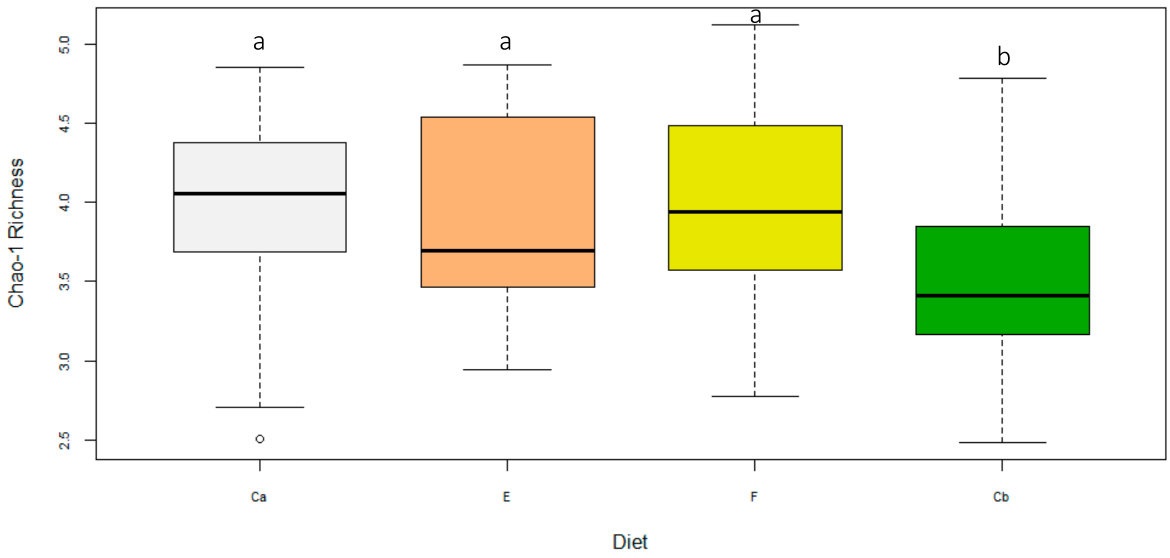

(**b**)

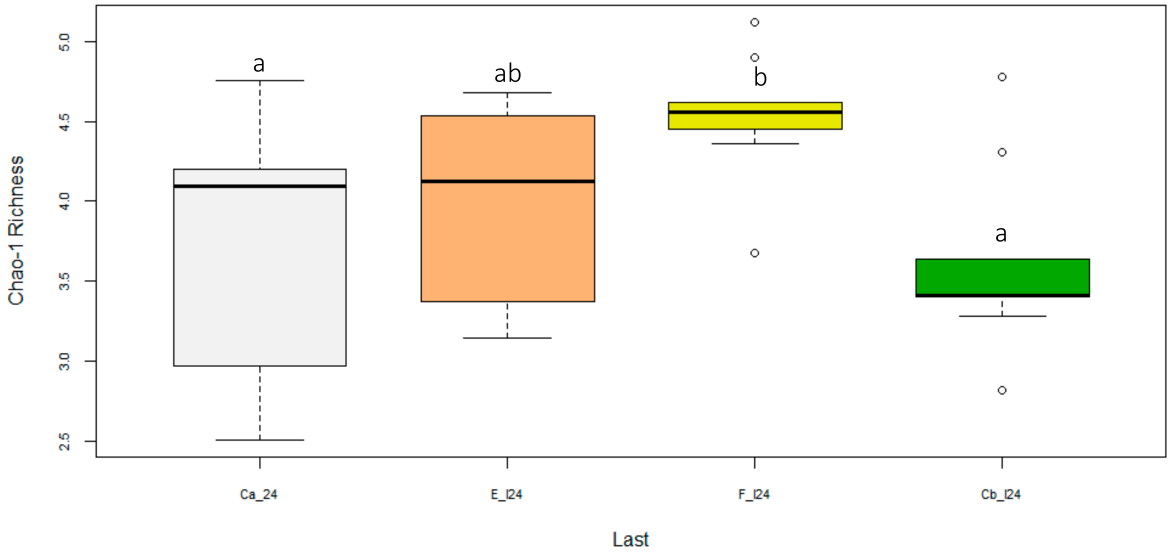

Figure S2. Alpha diversities of OTUs (mean ± SEM) in the intestine of rainbow trout for each diet (n = 9) based Chao-1 Richness at (**a**) pooled over four time points, and (**b**) D24 p.i. (last time point). Treatments included control+uninfected (Ca), erythromycin+infected (E), florfenicol+infected (F) and control+infected (Cb). Differing letters indicate significant difference ($p<0.05$).
